# Coccidioidomycosis and Blastomycosis: Endemic Mycotic Co-Infections in the HIV Patient

**DOI:** 10.14740/jocmr2036w

**Published:** 2014-12-29

**Authors:** Waqas Jehangir, Geeta Santoshi Tadepalli, Shuvendu Sen, Nina Regevik, Purnendu Sen

**Affiliations:** a Raritan Bay Medical Center, Perth Amboy, NJ, USA; b Ross University School of Medicine, Iselin, NJ, USA

**Keywords:** Coccidioidomycosis, Blastomycosis, Opportunistic infections, HIV patient, Dual infection, Non-endemic mycoses

## Abstract

Opportunistic fungal infections including aspergillosis species, candida species, and fusarium can be found in HIV-infected patients. Disseminated diseases due to endemic mycoses including histoplasmosis, coccidioidomycosis, and blastomycosis are all being reported among HIV patients who reside in the known endemic areas. However, in the non-endemic areas, or due to the rarity of these pathogens, it might be difficult to recognize these unfamiliar disease presentations. We report a patient with HIV who had dual infections with endemic mycotic infections of coccidioidomycosis and blastomycosis, as he had a brief stay in the endemic area.

## Introduction

*Coccidioides immitis* is a dimorphic fungus that is endemic to the Southwestern United States, mainly Arizona, California, and Texas, although recent cases of non-endemic *C. immitis* in areas such as Washington State have been reported [1-3]. Fungal infections usually presents as a mild flu-like illness, making it hard to differentiate from other differentials for respiratory illness [4]. Resolution typically takes several weeks with no further sequelae in a healthy individual. In an immunocompromised individual, however, dissemination can be rapid and fatal. For this reason, coccidioidomycosis is considered an AIDS-defining illness according to current CDC guidelines [5]. Extrapulmonary manifestations of *C. immitis* include skin, soft tissue, joints, and the meninges. A common presentation includes fever, shortness of breath, fatigue, night sweats, arthralgias, erythema nodosum, ocular hypersensitivity, meningeal irritation and unintentional weight loss. Given the varied presentation of this fungus, it should be considered as a strong differential diagnosis in cases of refractory pulmonary illness as well as meningitis [6].

On the other hand, *Blastomyces dermatitidis* is also a dimorphic fungus that is endemic to Central and Southeastern US, central New York State, Canada, India, Israel, Saudi Arabia, and Africa and is reportedly, one of the least common mycoses reported in HIV patients in North America [7]. It is usually asymptomatic in immuno-competent individuals but can cause considerable damage in the immunocompromised patient [7]. The manifestations of blastomycosis are varied and dependent upon individual immune reactions; its clinical presentation varies from asymptomatic, to mild flu-like symptoms, to severe ARDS. Given the varied clinical presentation of *B. dermatitis*, it should always be considered as a differential diagnosis in refractory pulmonary illnesses that do not respond to conventional antibiotic therapy, especially since it is now being reported in non-endemic areas such as upstate New York [8]. There is also some consideration about naming blastomycosis in an HIV-positive patient as an AIDS-defining illness [5]. Treatment depends on severity and spread of the disease. It usually encompasses an initial treatment with amphotericin B for the more severe manifestations followed with the use of an anti-fungal such as itraconazole once clinical improvement has been noted [9].

## Case Report

A 40-year-old man with past medical history significant for HIV non-compliant with highly active antiretroviral therapy (HAART) and asthma presented to ER following a 4-day history of watery diarrhea and vomiting. Four weeks prior to admission, he developed fever and malaise for which he was admitted. He was treated for tuberculosis and also received bactrim prophylaxis. Patient also presented with shortness of breath, loss of appetite, progressive loss of weight, and generalized body weakness; he also had recurrent attacks of pneumonia and diarrheal illness. Patient had multiple sexual partners, was a former smoker with a history of 60 pack years, an IV heroin abuser as well as a social drinker. Patient denied any recent travel outside of United States but had a brief stay of 2 weeks in Texas prior to admission. Patient was on anti-tuberculosis medication and bactrim.

At the time of presentation, blood pressure was 102/48 mm Hg, pulse rate was 102/min, respiratory rate was 16/min, and temperature was 99.4 °F with a saturation of 95% on room air. Patient was awake, alert, emaciated, and in mild respiratory distress. Mild bilateral enlargement of the parotid gland was noted, with multiple enlarged lymph nodes on both sides of the neck. Patient had tachycardia with decreased air entry bilaterally with dullness and crepitation noted at the basal portions of both lungs. Mild epigastric tenderness was appreciated without rigidity, guarding or organomegaly.

Laboratory investigations were as follows: WBC count 5,800/µL with PMN 76% and lymphocytes 11%, hemoglobin 11.5 g/dL, and platelets 310,000,000/µL. Chemistry was normal. The patient had a CD4 count of 4 and viral load 693,196 copies/mL. Sepsis workup was sent. Chest X-ray showed interstitial infiltrate with multiple nodular opacities in both lungs.

Patient was admitted to the floor with the diagnosis of chronic diarrheal illness, secondary to immunodeficiency and PCP pneumonia. Patient was started on IV fluids, bactrim and empirical antibiotics. At the same time, anti-tuberculosis medications were continued. On the third hospital day, he spiked a fever of 103.4 °F and developed severe respiratory distress. At this point, he was intubated and started on steroid therapy. Cultures were taken repeatedly for bacterial and for viral isolation. Despite treatment, patient’s condition progressively deteriorated and he later developed ARDS. He eventually went into cardiopulmonary arrest and expired on the eighth day of hospitalization.

**Figure 1:**
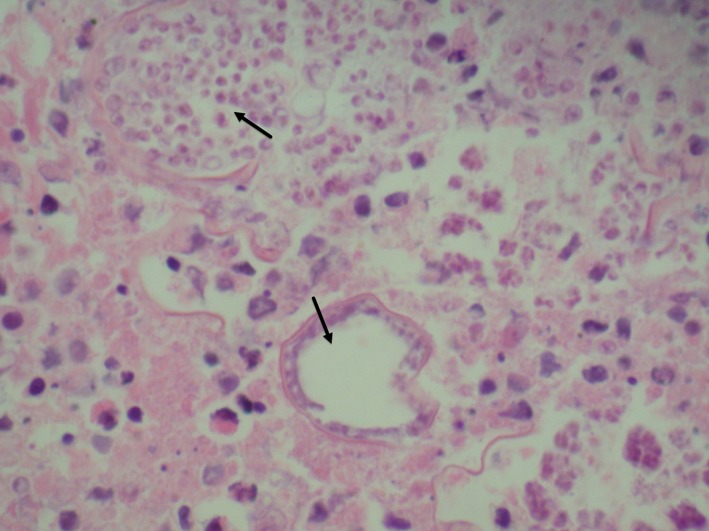
Lymph node showing extensive necrosis and numerous cysts containing numerous nuclei as well as the broad budding yeasts consistent with blastomyces.

**Figure 2:**
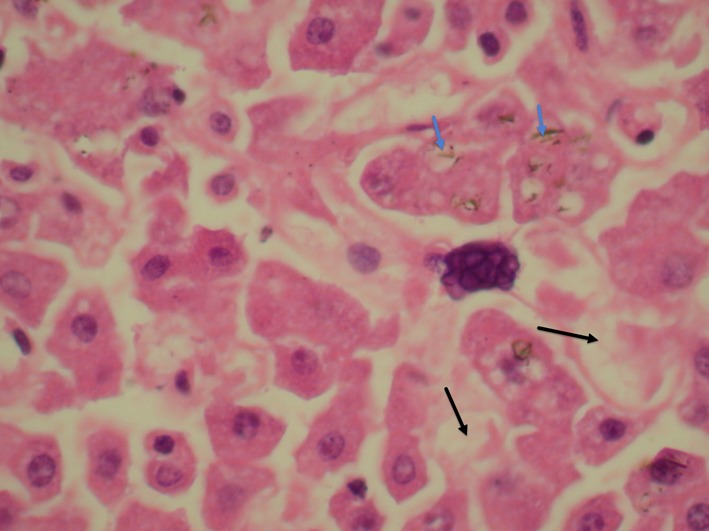
Liver showing few granuloma with central necrosis (black arrows) containing a large number of double refractile wall yeasts with occasional broad base budding consistent with blastomyces. Occasional yeasts are also seen engulfed within the giant cells (blue arrows).

**Figure 3:**
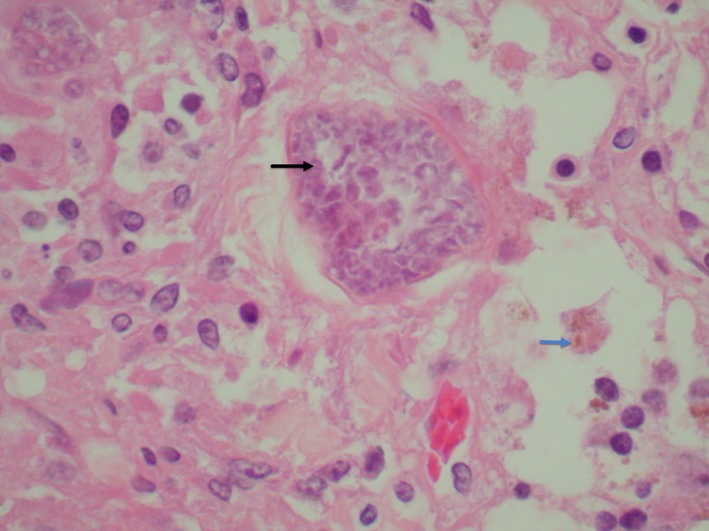
Thyroid gland granuloma containing numerous large yeasts with refractile wall and numerous nuclei (black arrows) and occasional broad base budding yeasts are identified consistent with blastomyces (blue arrows).

Ante-mortem blood cultures later reported positive for *C. immitis*. An autopsy was performed and showed a disseminated blastomycosis with superimposed coccidioidomycosis and milliary nodules in the lung. Histology showed multiple foci of necrosis with hemorrhages consistent with infarct. Multiple yeast organisms with broad budding yeast were identified and found to be consistent with blastomyces. There were multiple areas of partial calcification, particularly in the mediastinum and cervical area. Lung histology showed multiple nodular foci composed of necrotic centers within which there were numerous large cystic structures with thick retractile wall. Within these cystic structures were numerous, small nuclei and numerous yeasts with retractile walls and identifiable broad-based budding consistent with blastomyces. In addition, larger cysts containing round, large, endo-spherules, smaller cysts containing no spherules (immature), and a ruptured cyst consistent with coccidioidomycosis, were also found, as shown in Figures 1-3 respectively.

## Discussion

A variety of fungal infections are considered to be opportunistic in HIV-positive patients. There have been some individual cases of coccidioidomycosis, blastomycosis, and other opportunistic infections reported in the HIV-positive patient population. However, cases of dual infections of coccidioidomycosis and blastomycosis in the HIV-positive patient have been rare with less than 10 cases reported thus far [10].

There are many theories as to why exactly an HIV patient becomes susceptible to these particular fungal infections. Studies have shown that T cell-mediated immunity is critical to controlling and clearing *B. dermatitidis* and *C. immitis* infections [1, 11]. A failure of these mechanisms, as in the case of HIV, makes the organism more pathogenic, thereby leading to a more persistent and disseminated illness.

Furthermore, the fungi launch their own response on the already weakened immune system and weaken the intracellular killing mechanism, giving the fungi free reign within the body [12, 13]. The fungi escape immune surveillance and in doing so, lead to disseminated disease. Current treatment regimen with amphotericin B seems to be the most effective means of eliminating the fungi from the body and decreasing the incidence of disseminated and persistent illness [8, 14].

With regards to this case, a drastically low CD4 count coupled with a high viral load placed the patient in a significantly immunocompromised state. Factors further complicating his immune status were his non-compliance with HAART, an active tuberculosis infection, and recent travel to an endemic area known to cause opportunistic infections in an HIV-positive patient. The unique feature of this patient’s illness, however, was the presence of two disseminated opportunistic infections superimposed upon one another, leading to a more complicated illness course in our patient.

It is our hope that this case report brings light to the fact that there can be more than one causative organism masquerading as an opportunistic infection and that the clinician should be aware of this and guide their practice accordingly.
